# Using Structural Neuroimaging to Make Quantitative Predictions of Symptom Progression in Individuals at Ultra-High Risk for Psychosis

**DOI:** 10.3389/fpsyt.2013.00187

**Published:** 2014-01-29

**Authors:** Stefania Tognin, William Pettersson-Yeo, Isabel Valli, Chloe Hutton, James Woolley, Paul Allen, Philip McGuire, Andrea Mechelli

**Affiliations:** ^1^Department of Psychosis Studies, Institute of Psychiatry, King’s College London, London, UK; ^2^Wellcome Trust Centre for Neuroimaging, UCL Institute of Neurology, University College London, London, UK; ^3^Division of Experimental Medicine, Imperial College London, London, UK

**Keywords:** relevance vector regression, magnetic resonance imaging, cortical thickness, ultra-high risk, psychosis, symptom progression, prediction

## Abstract

Neuroimaging holds the promise that it may one day aid the clinical assessment of individual psychiatric patients. However, the vast majority of studies published so far have been based on average differences between groups, which do not permit accurate inferences at the level of the individual. We examined the potential of structural Magnetic Resonance Imaging (MRI) data for making accurate quantitative predictions about symptom progression in individuals at ultra-high risk for developing psychosis. Forty people at ultra-high risk for psychosis were scanned using structural MRI at first clinical presentation and assessed over a period of 2 years using the Positive and Negative Syndrome Scale. Using a multivariate machine learning method known as relevance vector regression (RVR), we examined the relationship between brain structure at first clinical presentation, characterized in terms of gray matter (GM) volume and cortical thickness (CT), and symptom progression at 2-year follow-up. The application of RVR to whole-brain CT MRI data allowed quantitative prediction of clinical scores with statistically significant accuracy (correlation = 0.34, *p* = 0.026; Mean Squared-Error = 249.63, *p* = 0.024). This prediction was informed by regions traditionally associated with schizophrenia, namely the right lateral and medial temporal cortex and the left insular cortex. In contrast, the application of RVR to GM volume did not allow prediction of symptom progression with statistically significant accuracy. These results provide proof-of-concept that it could be possible to use structural MRI to inform quantitative prediction of symptom progression in individuals at ultra-high risk of developing psychosis. This would enable clinicians to target those individuals at greatest need of preventative interventions thereby resulting in a more efficient use of health care resources.

## Introduction

The first full-blown psychotic episode is usually preceded by a prodromal phase which is characterized by a progressive decline in functioning and the emergence of attenuated psychotic symptoms. Individuals with these clinical features are said to be at ultra-high risk (UHR) for developing psychosis. Results from a recent meta-analysis suggest that about 18–36% of the UHR population will develop a psychotic disorder within 3 years from first clinical presentation (1). Thus, the study of the UHR population offers a window into the early stages of the illness under minimal influence of confounding factors such as medication and chronicity, and may inform the development of new early interventions aimed at delaying or preventing the onset of the illness.

Neuroimaging offers a promising translational tool for the characterization of brain abnormalities in individuals at UHR for psychosis; in particular, it has been suggested that neuroanatomical and neurofunctional measures could eventually be used to make individualized predictions of clinical outcome in this population. Consistent with this notion, a growing number of studies using structural Magnetic Resonance Imaging (MRI) have identified neuroanatomical differences between individuals at UHR who subsequently did and did not develop psychotic symptoms. Below we provide a brief overview of these studies, and then report the results of a novel investigation that examined whether structural MRI allows accurate quantitative predictions about symptom progression in individuals at UHR for psychosis.

Structural MRI has revealed a number of neuroanatomical differences at first clinical presentation between individuals who subsequently make transition to psychosis (UHR-T) and those who do not (UHR-NT). In whole-brain voxel-based morphometry (VBM) studies, UHR-T relative to UHR-NT subjects showed reduced gray matter (GM) volume of the inferior frontal cortex, medial and lateral temporal, anterior cingulate cortex (ACC), insular, inferior and superior frontal cortices (2), and reduced GM density of the left temporal lobe and right cerebellum (3). In addition, VBM studies employing a region of interest (ROI) approach indicated that individuals who subsequently make transition to psychosis show reduced volume in the left parahippocampal gyrus (4), the bilateral insula (5) and the left ACC (6), and increased volume of the pituitary gland (7, 8) and the hippocampus (9). A recent VBM investigation has also shown that, in individuals at UHR for psychosis, lower scores on a semantic fluency task are associated with reduced GM density in a distributed network including the right superior/middle temporal gyrus, the right insula, and the left ACC, suggesting that the combination of these two types of data could inform outcome prediction in this population (10).

Neuroanatomical alterations in psychosis may be expressed not only in terms of alterations in GM volume or density but also as changes in regional cortical thickness (CT) (11–15) and the degree of CT asymmetry (16). Consistent with this notion, UHR-T compared to UHR-NT have been found to show cortical thinning of the ACC (17). However, a few subsequent ROI studies did not find any significant differences in CT between individuals who subsequently did and did not make conversion to psychosis (9, 18–23).

While the above studies used a cross-sectional design, a number of investigations have employed a within-subject design to examine the neuroanatomical changes that occur in individuals at UHR around the time of illness onset. These studies have reported progressive reductions in the GM volume of the orbitofrontal and cerebellar cortices (2, 24), fusiform and parahippocampal cortices and cingulate gyrus (24); superior frontal, inferior temporal, superior parietal cortices, and precuneus (2) in UHR-T compared to UHR-NT. In addition, using an ROI approach, within-subject studies have found greater progressive reductions in the GM volume of the insula (5), planum polare, planum temporale, and caudal region (22) in UHR-T compared to UHR-NT (22). Using cortical pattern matching techniques, Sun and colleagues (25) have also revealed volumetric reductions in the prefrontal cortex in UHR-T compared to UHR-NT (25). With respect to CT, the only longitudinal study published so far has reported progressive thinning of the anterior cingulate, precuneus, and temporal-parieto-occipital cortex in UHR-T compared to UHR-NT and healthy controls (26).

A small fraction of studies of the UHR population have investigated white matter (WM) abnormalities associated with transition to psychosis using either VBM or diffusion tensor magnetic resonance imaging (DTI). With respect to VBM, only two studies have investigated WM abnormalities in UHR subjects as a function of clinical outcome (26, 27). In the first study, UHR-T subjects showed increased WM volume in the left frontal lobe and a progressive decrease in the left fronto-occipital fasciculus (27). In the second study, UHR-T subjects showed a decrease in total WM volume relative to healthy controls but not relative to UHR-NT, in addition to which the comparison between UHR-NT and controls was also not significant (26). With respect to DTI, a large number of studies have compared individuals at UHR against healthy controls (28–34) but only three of them have subdivided the UHR group according to clinical outcome (30–32). One of these studies revealed that UHR-T had lower fractional anisotropy (FA), an index of WM integrity, at baseline compared to healthy controls in the medial frontal region (30). In addition, UHR-T had lower FA in the WM lateral to the right putamen and in the left superior temporal gyrus but higher FA in the left posterior temporal WM, compared to UHR-NT (30). Finally, in UHR-T, the FA in the left middle temporal lobe was negatively associated with the severity of positive symptoms (30). The remaining two studies reported no cross-sectional differences in WM integrity between UHR-T and UHR-NT (31, 32). However, Carletti and colleagues (31) reported a progressive reduction of left frontal WM in UHR-T which was not evident in UHR-NT (31).

Taken collectively, the above studies provide evidence for differences in brain structure between individuals at ultra-high risk for psychosis who subsequently do and do not develop the illness, particularly in the prefrontal and temporal cortices. These studies, however, each reported significant effects only at a group level, whereas clinicians treating psychosis have to make decisions about the individual in front of them. Because effects that are significant at a group level do not necessarily permit accurate inferences about individuals, the translational potential of the above findings for everyday clinical practice is unclear. In addition, these studies were conducted using a standard univariate analytical approach in which each voxel is considered independently. This approach is well suited to detect effects that are robust and localized; however, it is not very sensitive to differences that are subtle and highly distributed across the brain. For these reasons, an increasing number of recent studies of psychiatric disorders have adopted an alternative approach based on multivariate machine learning methods (35, 36). A key benefit of multivariate machine learning methods is that they allow one to make predictions that are specific to a given individual, rather than providing an average estimate for a group. This greatly increases the likelihood that the results can be translated into a tool that is useful in a real world clinical setting. A further benefit of multivariate machine learning methods is that they take into account the inter-relationship between different measures (e.g., GM volume across different voxels), and therefore are better suited for detecting subtle and spatially distributed patterns of alteration. The vast majority of multivariate machine learning studies of psychiatric disorders published so far have been limited to categorical decisions such as whether an individual belongs to a patient or control group; whether an individual will respond to treatment or not; or whether an individual will develop a disorder or not (35). Within this context, studies of the UHR population employing multivariate machine learning methods have typically focused on prediction of clinical outcome in terms of transition/non-transition to psychosis. For example, Koutsouleris and colleagues (37) demonstrated that a distributed network of abnormalities in GM volume allows prediction of subsequent transition to psychosis with an accuracy of 82% (37). This notable finding was replicated in an independent cohort by a subsequent investigation (38). However, follow-up studies of individuals at UHR have shown substantial heterogeneity in symptom progression both among those who develop psychosis and those who do not (39, 40). For instance, a recent investigation showed that about 75% of those individuals who do not develop psychosis present with symptoms remission after 3 years while the remaining 25% are still showing sub-threshold symptoms (40). In addition, even those individuals at UHR who show full or partial remission of positive symptoms remain at a lower level of functioning compared to non-psychiatric comparison individuals (41). Another study reported that only 30% of those individuals who do not develop psychosis experience a full symptomatic and functional recovery (42). Despite the high degree of heterogeneity in clinical outcome beyond and above transition of psychosis, none of the multivariate machine learning studies of the UHR population published so far have focused on quantitative changes in symptomatology.

Here we sought to expand the existing literature by investigating the potential of structural MRI for predicting the course of clinical symptomatology at 2-year follow-up in individuals at ultra-high risk for psychosis using Relevance Vector Regression (RVR) (43). The advantage of RVR relative to other multivariate machine learning techniques, such as Support Vector Machine (35), is that it allows the quantitative prediction of a variable of interest (e.g., a patient’s score on a clinical scale) at individual level, without the need for a discrete categorical decision (e.g., patients vs. controls). In recent years, RVR has been successfully used in several neuroimaging studies of healthy people (44, 45) and patients with psychiatric (46, 47) or neurological disorders (48). We therefore hypothesized that the application of RVR to neuroanatomical data, particularly GM volume and CT, would allow quantitative prediction of symptom progression at individual level with statistically significant accuracy.

## Materials and Methods

### Subjects

The total sample consisted of 40 subjects at ultra-high risk for psychosis (UHR), recruited at first presentation from consecutive referrals to the Outreach and Support in South London (OASIS) service in London, UK (49). OASIS is a clinical service located in Lambeth, South London, that offers treatment to individuals between 14 and 35 years of age who meet the ultra-high risk criteria for psychosis. Individuals at ultra-high risk for psychosis were identified based on the Personal Assessment and Crisis Evaluation (PACE) criteria (50).

Subsequent to MRI scanning, the UHR subjects were monitored for at least 2 years. Over the 2-year follow-up, 7 UHR individuals developed psychosis (UHR-T) and the remaining 33 did not (UHR-NT). Transition to psychosis during the follow-up period was established according to the Diagnostic and Statistical Manual of Mental Disorders, Fourth Edition (DSM-IV) criteria based on clinical consensus between at least two experienced psychiatrists. Most of the UHR group (31/44; 70%) were naïve to antipsychotics at the time of scanning; the remaining 13 (30%) had been exposed to antipsychotics for an average of 9.7 weeks (SD = 13.3).

### Socio-demographic and clinical measures

Socio-demographic measures included age, gender, and years of education. Clinical symptoms were assessed in all participants at the time of scanning and at 2-year follow-up using the Positive and Negative Syndrome Scale (PANSS) (51). Symptoms in the UHR participants were also assessed using the Comprehensive Assessment of At-Risk Mental States (CAARMS) (50). Socio-demographic and clinical variables were analyzed using Student’s *t*-test for continuous data and a chi square test for ordinal data. These statistical analyses were performed using the Statistical Package for the Social Sciences 19.0 (SPSS 19.0 for Windows, Chicago, IL, USA).

### Acquisition of neuroanatomical data

Neuroanatomical images were acquired using a 1.5-T GE NV/I Signa LX Horizon system (General Electric, Milwaukee, WI, USA) at the Center for Neuroimaging Sciences, King’s College London. T1-weighted Inversion Recovery Spoiled Gradient structural images were acquired with the following acquisition parameters: TE = 5200 ms, TR = 15900 ms, flip angle = 20°, field of view = 220 mm × 176 mm, slice thickness = 1.5 mm, number of slices = 124, image matrix = 256 × 256 × 124.

### Analysis of neuroanatomical data

The analysis of the MRI data comprised of three main components. Firstly, the unified segmentation procedure (52) implemented in SPM8^1^ was used to segment all the images into GM, WM, and cerebrospinal fluid (CSF) partitions. We then pre-processed the images using two alternative approaches that allowed us to extract information on GM volume and CT respectively. Secondly, we used multivariate RVR (43) as implemented in the Pattern Recognition for Neuroimaging Toolbox^2^ (PRoNTo). Thirdly, we performed a standard univariate analysis as implemented in Statistical Parametric Mapping (SPM8) software. Below we describe each component in more detail.

#### Creation of voxel-based gray matter volume maps

A fast diffeomorphic image registration algorithm (DARTEL) was used to warp the GM partitions into a new study-specific reference space with an isotropic spatial resolution of 1.5 mm^3^ (53–55). The warped GM partitions were then affine transformed into the MNI space. An additional “modulation” step (56) was used to scale the GM probability values by the Jacobian determinants of the deformations to ensure that the total amount of GM in each voxel was conserved after the registration. As a final step the GM probability values were smoothed using a 8-mm FWHM Gaussian kernel.

#### Creation of voxel-based cortical thickness maps

A voxel-based Laplacian method (57, 58) was used to create a voxel-based cortical thickness (VBCT) map for each subject using the GM, WM, and CSF partitions created in the segmentation step. The resulting VBCT maps contained CT values within voxels identified as GM and zeros outside the cortex and were saved in the native space of the input images (0.5 mm^3^ resolution). Each VBCT map was warped into the new DARTEL reference space by applying the corresponding subject-specific deformation field and resampled to an isotropic voxel size of 1.5 mm^3^. The warped images were then scaled by the Jacobian determinant of the deformation and smoothed with a 6-mm Gaussian kernel. The same warps, modulation and smoothing were also applied to a binary mask created from each original VBCT map. Subsequently the warped, scaled, and smoothed VBCT maps were divided by the corresponding warped, scaled, and smoothed mask. The effect of this procedure was to project the Gaussian smoothing kernel applied to the warped images, into the native space of the subject while preserving the CT value over a region the size of the smoothing kernel.

#### Multivariate RVR analyses

We examined the relationship between brain structure and changes in PANSS total score from baseline to 2 years follow-up using multivariate RVR as implemented in PRoNTo (see text footnote 2) running under Matlab (Mathworks, 2010 release) (59). This method has been described elsewhere (47). In brief, RVR is a sparse kernel learning multivariate regression method set in a fully probabilistic Bayesian framework. Under this framework, a zero-mean Gaussian prior is introduced over the model weights, governed by a set of hyperparameters – one for each weight. The most probable values for these hyperparameters are then iteratively estimated from the training data, with sparseness achieved due to the posterior distributions of many of the weights peaking sharply around zero; those training vectors associated with non-zero weights are referred to as “relevance” vectors. The optimized posterior distribution over the weights can then be used to predict the target value (e.g., PANSS score) for a previously unseen input vector (e.g., CT map) by computing the predictive distribution [for a more in-depth and detailed description see Tipping (43)].

In the current study, the input vectors (i.e., each subjects CT map) were mean centered using the training data, and an estimate for the model’s generalizability obtained via leave-one-out cross validation, indexed using the Pearson correlation coefficient and mean square error (MSE) between actual and predicted difference between baseline and follow-up on PANSS total scores. The significance of both the correlation coefficient and the MSE score was estimated using a permutation test whereby the input-target data were randomly paired and the RVR re-run 1000 times. This created a distribution of correlation and MSE values reflecting the null hypothesis that the model did not exceed chance. The number of times the permuted value was greater than (or with respect to MSE values, less than), or equal to, the true value, was then divided by 1000 providing an estimated *p*-value for both the correlation coefficient and observed MSE. For ease of visualization, a table was also created using an arbitrary 70% threshold for all successful RVR derived weight maps, showing those regions with weight vector values in the upper, and lower, 30% of the absolute maximum weight vector values across all regions. These values represent the relative contribution of each voxel to the regression function, in the context of every other voxel.

#### Univariate SPM analyses

We also examined the relationship between brain structure and changes in PANSS total score from baseline to follow-up using a standard, univariate approach. A multiple regression model was performed in SPM8 software to identify any voxels in the GM volume and CT maps respectively that showed a significant association with PANSS total scores. Statistical inferences were made at *p* < 0.05 [corrected for multiple comparisons using Family-Wise Error (FWE)]. For completeness, when no significant effects were found, we also examined trends at *p* < 0.001 uncorrected.

## Results

### Socio-demographic and clinical characteristics

Socio-demographic and clinical variables are reported in Table 1 for all participants as well as for the sub-groups that did and did not make transition to psychosis separately. It can be seen that, on average, participants showed clinical improvement at follow-up relative to baseline (*t* = −2.555; *p* = 0.015; df = 39). Examination of the subject-specific scores revealed that 26 individuals improved, 3 remained stable, and 11 worsened over the 2-years follow-up time. No significant association were found between the change in PANSS total scores from baseline to follow-up and antipsychotic medication (*t* = −0.269, df = 38, *p* = 0.789).

**Table 1:** **Demographic and clinical variables by group**.

	Groups			Group comparison
	UHR (*N* = 40)	UHR-NT (*N* = 33)	UHR-T (*N* = 7)	
Age (years)	23.90 (4.50)	24.06 (4.61)	23.14 (4.18)	*t* = 0.48, *p* = 0.63 df = 38
*N* male/female	25/15	20/13	5/2	χ^2^ = 0.29, *p* = 0.59
Years of education	12.82 (2.31)	12.88 (2.31)	12.50 (2.51)	*t* = 0.36, *p* = 0.72, df = 36
PANSS total baseline	53.30 (14.95)	50.27 (12.02)	67.57 (19.85)	*t* = −3.06, *p* = 0.004, df = 38
PANSS total follow-up	46.50 (13.34)	43.61 (10.25)	60.14 (18.27)	*t* = −3.34, *p* = 0.002, df = 38
Difference PANSS follow-up – baseline	−6.80 (16.83); *t* = −2.555; *p* = 0.015; df = 39	6.67 (15.16)	7.43 (24.78)	*t* = −0.10, *p* = 0.91, df = 38

*Data reflect mean (and standard deviation). df, Degrees of freedom; PANSS, positive and negative syndrome scale*.

### Multivariate RVR analysis

**Figure 1:**
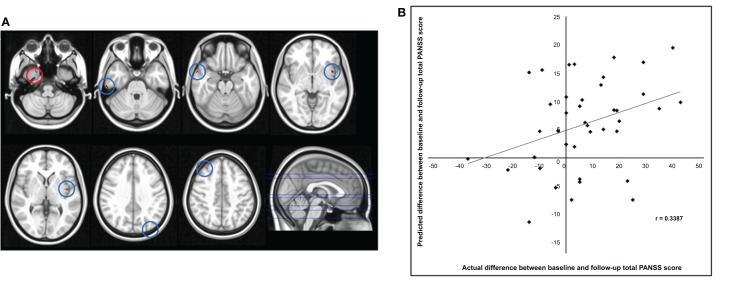
**(A)** Red/Blue circles show voxels with a weight score in the top/bottom 30% of the maximum (range −0.011608 to –0.026588). Axial Slices (MNI) Left-Right: −65, −49, −40, −23, −17, 16, 25. **(B)** Scatter plot showing the predicted difference between baseline and follow-up total PANSS score for each subject derived from their cortical thickness data using RVR vs. the actual difference.

The application of RVR to whole-brain CT images allowed quantitative prediction of symptom progression with statistically significant accuracy (correlation = 0.34, *p*-value = 0.026; Mean Squared-Error = 249.63, *p*-value = 0.024, see Figure 1). The use of an arbitrary threshold corresponding to the top, or bottom, 30% of the maximum weight vector score showed that the prediction appeared to be based on a distributed pattern of CT including, in particular, the left insular cortex and lateral and medial regions of the right temporal cortex (see Table 2; Figure 1). In contrast, the application of RVR to the whole-brain GM volume images did not allow accurate prediction of symptoms progression (correlation = 0.14, *p*-value = 0.627; Mean Sum of Squares = 369.50, *p*-value = 0.621).

**Table 2:** **Neuroanatomical regions with a weight vector score in the top and in the bottom 30% of the maximum weight vector score across all regions for the cortical thickness based RVR used to accurately predict the difference between baseline and follow up total PANSS score**.

Region	Number of voxels	MNI coordinate (*x, y, z*)	*w_i_*
**REGIONS WITH POSITIVE *w_i_* SCORES**			
Right Temporal Fusiform Cortex	27	28.5, −9, −46.5	0.0266
Right Temporal Pole	8	25.5, 6, −48	0.0213
**REGIONS WITH NEGATIVE *w_i_* SCORES**			
Right Temporal Pole	29	58.5, 6, −22.5	0.0115
Left Insular Cortex	26	−39, 6, −4.5	0.0112
	20	−42, −6, −1.4	0.0116
Right Parahippocampal Gyrus (anterior division)	9	10.5, −12, −21	0.00954
Right Inferior Temporal Gyrus (posterior division)	7	60, −27, −30	0.00882

*MNI, Montreal Neurological Institute; RVR, relevance rector regression; PANSS, positive and negative syndrome scale; ***w_i_***, weight vector score indicating the relative contribution of each voxel to the regression function*.

****w_i_*** and MNI coordinates refer to the peak weight vector score in each cluster*.

### Univariate SPM analysis

Whole-brain analysis of the GM volume and CT data did not detect any regions that showed a significant positive or negative association with the change in PANSS total scores from baseline to follow-up at *p* < 0.05 (FWE corrected). With a less conservative statistical threshold (*p* < 0.001 uncorrected), we detected a number of regions showing a positive association with the change in PANSS total scores. With respect to GM volume, the right middle frontal gyrus (MNI coordinates: 39, 15, 37.5; *p* = 0.929; *z*-score = 3.266) was associated with changes in PANSS scores. With respect to CT, the right inferior parietal lobule (MNI coordinates: 61.5, −34.5, 25.5; *p* = 0.802; *z*-score = 3.659), left cingulate gyrus (MNI coordinates: −9, 1.5, 46.5; *p* = 0.986; *z*-score = 3.340), right middle temporal gyrus (MNI coordinates: 49.5, −63, 4.5; *p* = 0.992; *z*-score = 3.295) and left insula (MNI coordinates: −34.5, −15, 16.5; *p* = 0.998; *z*-score = 3.197) were associated with changes in PANSS scores.

## Discussion

At present it is difficult to use clinical data acquired at first clinical presentation to predict subsequent progression of symptoms in individuals at UHR for psychosis. This prevents the selective delivery of potentially preventative interventions to those who are most likely to develop persistent symptoms. Recent studies have shown that the application of multivariate machine learning methods to structural neuroimaging data allows accurate categorical prediction of which individuals at UHR will and will not make transition to psychosis (37). However, as discussed in the introduction, within the UHR population there is a substantial heterogeneity in symptom progression above and beyond transition to psychosis (40–42). We therefore examined for the first time whether it is possible to use neuroanatomical information to make accurate quantitative predictions of symptom progression in individuals at UHR. Our results indicate that the application of RVR to whole-brain CT MRI data allows quantitative prediction of symptom progression (i.e., both the magnitude and direction of change for each individual) at 2-year follow-up with statistically significant accuracy. To our knowledge, this is the first evidence that neuroimaging techniques may inform the clinical assessment of UHR individuals by allowing quantitative estimation of the course of clinical psychopathology. In contrast, GM volume did not allow accurate prediction of symptoms progression at individual level despite two previous reports that this information allows categorical prediction of transition to psychosis (37, 38).

What is the implication of our differential finding for CT and GM volume? GM volume is thought to depend on local CT as well as cortical folding and gyrification (i.e., cortical surface area), while CT does not include measures of local surface (57). A recent investigation has also shown that CT and cortical surface area are genetically and phenotypically independent, and that regional GM volume is more closely related to the latter than the former (60). It follows that the two approaches provide complementary information, and that one can be more or less than the other depending on the nature of the neuroanatomical changes being examined. In the context of our investigation, the fact that symptom progression was predicted by CT but not GM volume indicates that changes in symptomatology are specifically associated with differences in CT as opposed to cortical folding and gyrification.

Examination of the regions that provided the greatest contribution to prediction of symptom progression identified specific areas amongst others traditionally associated with schizophrenia, namely the right temporal fusiform cortex, the right temporal pole, the right parahippocampal gyrus, the inferior temporal gyrus, and the left insular cortex (see Table 2; Figure 1). The temporal fusiform cortex and temporal pole have been reported to show CT differences over time between UHR-T and controls but not between UHR-NT and controls (26). The temporal pole is thought to be implicated in different cognitive functions such as emotion, attention, behavior, and memory (61). In people with schizophrenia, abnormalities in this region have been associated with a range of clinical symptoms including, amongst others, auditory hallucinations and thought disorder (62, 63). The temporal fusiform cortex plays a central role in facial configuration processing in the healthy brain (64). Deficits in this domain have been recently reported in the UHR population, and may be one of the factors that underlie social dysfunction in schizophrenia (65). The parahippocampal gyrus has also been reported to show reduced thickness both in the UHR (20, 66) and first episode psychosis (67). Specifically, this area has been identified as a site of robust structural and functional alteration in individuals at ultra-high risk for psychosis (68) and those who have developed the disorder (69, 70). The right inferior temporal gyrus volume has also been reported as progressively reduced overtime in UHR-T compared to UHR-NT (2). Finally the left insular cortex plays a key role in emotional regulation, which is typically altered in psychosis, and has been found to show reduced volume in UHR-T compared to UHR-NT (2, 5).

While the results of our investigation provide further evidence for the implication of the above regions in schizophrenia, it should be noted that in multivariate methods an individual region may display high discriminative power due to two possible reasons: (i) a difference in volume between groups in that region; (ii) a difference in the correlation between that region and other areas between groups. Thus, the regions identified in our investigation should be interpreted as parts of a spatially distributed pattern rather than as independent areas. In addition, it should be noted that these regions were identified using an arbitrary threshold of 30% based on previous studies (71, 72), and that prediction of symptom progression was to some extent informed by all voxels in the brain since no feature extraction was employed.

In contrast to the results obtained using RVR, the univariate analysis of the structural MRI data, in which each voxel is considered as a spatially independent unit, did not detect any regions that showed a significant association with progression of symptoms after correction for multiple comparisons. This supports the idea that multivariate methods such as RVR are more sensitive to the subtle and spatially diffuse alterations typically observed in psychiatric disorders, and therefore may be better suited to the possible development of clinical diagnostic tools, than standard mass-univariate techniques (73).

The present study has four main limitations. Firstly, the number of subjects included in the study was relatively small and therefore the generalizability of the results is unclear. Multi-center studies would be needed in order to better characterize the predictive value of structural neuroimaging for predicting symptom progression in real-life clinical practice. Secondly, 30% of our participants had been exposed to antipsychotic medication which might have influenced our results for instance by resulting in changes in brain structure while also influencing symptom progression. Nevertheless, as we report in the Results, we found no evidence for an association between antipsychotic medication at first clinical presentation (i.e., yes/no) and progression of illness. Thirdly, there are a number of potential sources of individual variability in symptom progression that were not included in our statistical model; these include, for example, socio-demographic variables such as age, gender, and ethnicity, and treatment course variables such as life events and psychosocial interventions during the follow-up period. We expect that the integration of this information within the same statistical model would improve prediction of symptom progression. Fourthly, in the present investigation we examined the predictive value of gray rather than WM as the former could be estimated more accurately than the latter. However, given the number of studies reporting an association between WM integrity and clinical outcome in the UHR population (26, 27, 30–32), it would be interesting to use DTI scans in future studies.

In conclusion, the results of the present study provide proof-of-concept that it might be possible to use structural neuroimaging to inform quantitative prediction of subsequent progression of symptoms in individuals at UHR for psychosis. This would enable clinicians to target those individuals at greatest need of preventative interventions thereby resulting in a more efficient use of health care resources. It should be noted, however, that daily clinical practice often requires clinicians to make prompt treatment decisions, and delaying the decisional process in order to acquire and analyze structural neuroimaging data could be impractical and potentially harmful to a patient. A possible solution would be the development of a practical and flexible analytical tool for clinical use that does not require the manual implementation of a lengthy pipeline. In addition, it is likely that the use of structural neuroimaging in everyday clinical practice would ultimately require a greater accuracy of prediction than that found in the present study. Such accuracy might be improved, for example, by combining structural neuroimaging with other types of data, an integrative approach which was successfully applied to an investigation of mild cognitive impairment (74).

## Author Contributions

Stefania Tognin performed the analyses and prepared the first draft of the manuscript; William Pettersson-Yeo assisted with data analyses and prepared the first draft of figure and tables. Isabel Valli and James Woolley contributed with data collection; Chloe Hutton provided the VBCT toolbox and assisted with data analyses. Andrea Mechelli designed the research study. All the co-authors contributed to the critical revision of the manuscript.

## Conflict of Interest Statement

The authors declare that the research was conducted in the absence of any commercial or financial relationships that could be construed as a potential conflict of interest.

## Footnotes

^1^http://www.fil.ion.ucl.ac.uk/spm

^2^http://www.mlnl.cs.ucl.ac.uk/pronto/
